# High Sensitivity of the *rpiAB Escherichia coli* Mutants to Antibiotics Is Associated with the Participation of the *deo*-Operon in a Hypothetical Ribose-5-Phosphate Futile Cycle

**DOI:** 10.3390/cells15191740

**Published:** 2026-09-24

**Authors:** Tatyana A. Seregina, Rustem S. Shakulov, Svetlana A. Sklyarova, Alexander S. Mironov

**Affiliations:** Engelhardt Institute of Molecular Biology, Russian Academy of Science, 119991 Moscow, Russia; rshakulov@yandex.ru (R.S.S.); sklyarovasveta@yahoo.com (S.A.S.); alexmir_98@yahoo.com (A.S.M.)

**Keywords:** nucleoside catabolism, *deo*-operon, salvage pathway, futile cycle, ribose-5-phosphate, ribose-5-phosphate isomerases RpiA and RpiB, antibiotics

## Abstract

Previously, we demonstrated that disruption of ribose-5-phosphate biosynthesis in the pentose phosphate pathway increases the sensitivity of *E. coli* bacteria to a broad spectrum of antibiotics, and this sensitivity can be suppressed by deletion of the *deoB* gene. In this study, we showed that antibiotic sensitivity can also be suppressed by the deletion of the *apt* gene, which, along with the *deoBD* genes, constitutes the salvage pathway for purine base uptake. We propose that in the *rpiAB* mutant, these genes, together with the *prs* gene responsible for phosphoribosyl pyrophosphate (PRPP) synthesis, participate in a futile cycle, converting ATP to AMP and pyrophosphate (PPi). The data obtained on the genetic complementation of deletions of the *apt* and *deoBD* genes, combined with restored ATP levels in the *rpiAB* mutant cells, support the model of the hypothetical futile cycle of ribose-5-phosphate.

## 1. Introduction

Recent research on overcoming the problem of antibiotic resistance in clinical and socially important pathogens has focused on the non-specific effects of antibiotics on bacterial metabolism in an attempt to find ways to reduce the adaptive potential of the cell to drug action [1,2,3]. Previously, we have demonstrated that the mutational damage to the biosynthesis of ribose-5-phosphate (R5P) in the pentose phosphate pathway (PPP) increases the sensitivity of *E. coli* bacteria to a wide range of antibiotics [4,5]. This sensitivity was associated with increased R5P levels in cells and was suppressed by mutations that activate R5P metabolism or limit its synthesis. Enhancing the synthesis of LPS cell wall components associated with the consumption of sedoheptulose-7-phosphate (S7P), which originates from R5P, as well as restriction creation of R5P through the inactivation of the DeoB enzyme, which controls the conversion of ribose-1-phasphate (R1P) to R5P during ribonucleoside catabolism, were factors suppressing the phenotype of supersensitivity of the *rpiAB* mutant to antibiotics and exogenous ribose [5]. Previous studies suggest that nucleoside catabolism is another source of R5P in the cell, capable of significantly altering its levels under conditions of limited R5P metabolization through the non-oxidative branch of the PPP into glycolysis [4].

The process of nucleoside degradation in *E. coli* is performed by enzymes whose genes constitute the *deo*-operon, which has a complex regulation [6,7]. The *deo*-operon consists of two functional parts. The first part is responsible for the catabolism of pyrimidine deoxyribonucleosides and is represented by the *deoC* gene encoding deoxyriboaldolase and *deoA* encoding thymidine phosphorylase, which are under the control of promoters P1 and P2 (Figure 1). The genes *deoB*, which encodes phosphopentomutase, and *deoD*, which encodes purine nucleoside phosphorylase, are involved in the catabolism of purine nucleosides and constitute the second part of the operon under the control of its own promoter P3 (Figure 1) [8].

It should be noted that phosphopentomutase encoded by *deoB* is a non-specific enzyme with respect to deoxyribose-1-phosphate (dR1P) and R1P, possessing a lower Km for dR1P (13 µM) compared to the Km for R1P (43 µM) [9]. Thus, the *deo*-operon is commonly viewed primarily as a pathway for deoxyribonucleoside degradation [7,10]. However, the presence of the P3 promoter in front of the *deoBD* genes, which is not controlled by the primary repressors of the *deo*-operon DeoR and CytR with the participation of the inducers deoxyribose-5-phosphate and cytidine [11,12], respectively, implies that the second part of the operon predominantly degrades purine ribonucleosides. Additionally, the transcription from the P3 promoter can be induced by ribosides inosine and guanosine [8].

This work continues a series of investigations focused on the impact of R5P metabolism on the sensitivity of *E. coli* cells to antibiotics. Here we study in detail the influence of other *deo*-operon genes on the antibiotic sensitivity of the *ΔrpiAB* mutant. We found that along with the *deoB* gene, the product of the *deoD* gene, responsible for degradating purine ribonucleosides into purine nucleobases and ribose-1-phosphate, is also required for antibiotic sensitivity. It is known that these nucleobases can be reutilized through a salvage pathway due to the activity of phosphoribosyltranspherases, which phosphorylate nucleobases to mononucleotides [13]. Therefore, we studied the possible role of phosphoribosyltranspherase gene *apt* in the antibiotic sensitivity of the *ΔrpiAB* mutant. Remarkably, the second component of the salvage pathway is phosphorybosyl diphosphate (PRPP), which originates from R5P via the activity of the *prs* gene [14]. Thus, in this work we investigate the antibiotic sensitivity of the *ΔrpiAB* mutant as a function of the metabolic interaction between the *deo*-operon and a salvage pathway enzyme, which may be considered as a futile cycle.

## 2. Materials and Methods

### 2.1. Bacterial Strains

The bacterial strains of *E. coli* and the plasmids used in this work and their genotypes are presented in Table 1.

Triple mutants AM4011, AM4012 and AM4016 were constructed as described previously [5]. The combination of deletions in the *deoD*, *deoC*, and *apt* genes with mutations in the ribose-5-phosphate isomerase genes *rpiAB* was carried out by transduction with phage P1 using strains from the KEIO collection [15]. The kanamycin cassette was removed from the resulting strains using the helper plasmid pCP20 [16] resulting in the formation of the corresponding deletions *∆deoD*, *∆deoC* and *∆apt.* The presence of deletions was confirmed by PCR experiments. The obtaining of an *rpiAB* mutant with a deletion of the promoter region P1-P2 of the *deo*-operon before the *deoC* gene was implemented using the Datsenko-Wanner method described in [16]. All primers used in this study are presented in Table A1.

### 2.2. Media and Growing Conditions

Bacterial cultures were grown in complete liquid Luria–Bertani (LB) medium (L3022, Sigma-Aldrich, St. Louis, MO, USA) at 37 °C on a thermostatted shaker (200 rpm). LB with the addition of 1.5% bacteriological agar was used as a solid medium. If necessary, the following were added to the medium: nalidixic acid, moxifloxacin, gentamicin, erythromycin, rifampicin, kanamycin, chloramphenicol, adenosine, guanosine, deoxyadenosine and deoxyguanosine in the concentrations given under the corresponding figures in the Results section. All reagents used in the work were produced by Sigma-Aldrich, St. Louis, MO, USA, unless otherwise stated.

### 2.3. Generation of Growth Curves

Growth curves were plotted using an automatic Bioscreen C device (Oy Growth Curves Ab Ltd., Turku, Finland) as described in [5]. Overnight cultures of bacteria grown at 37 °C in LB medium were diluted 100 times, placed in the wells of the Bioscreen C device platform and grown at 37 °C with maximum shaking. OD_600_ values were recorded automatically at certain time intervals. Each experiment was carried out in triplicate and the average values were used to construct bacterial growth curves.

### 2.4. Determination of Bacterial Sensitivity to Antibiotics

Time-killing experiments under conditions of high doses of antibiotics were carried out in accordance with the requirements of the Clinical and Laboratory Standards Institute [17]. Bacterial cultures grown to mid-logarithmic phase (OD_600_ ≈ 0.5) were incubated in the presence of antibiotics at the indicated concentrations for 30 min. A series of 10-fold dilutions were prepared and plated on solid LB medium for colony counting. MICs for the strains used in this study are presented in Table A2. Colony formation ability was assessed by the microdilution method as described in [5].

### 2.5. Genetic Complementation of Deletions of deoB, deoD and apt

The antibiotic susceptibility phenotypes of the triple mutants *ΔrpiAB ΔdeoB*, *ΔrpiAB ΔdeoD*, and *ΔrpiAB Δapt* were tested by genetic complementation using expression vectors containing the wild alleles of the *deoB*, *deoD*, and *apt* genes. Structural regions of the *deoD* and *apt* genes were cloned into the pUC19 vector at the BamHI and EcoRI restriction sites as described previously [4]. The resulting plasmids were transformed into cells of the *ΔrpiAB ΔdeoB*, *ΔrpiAB ΔdeoD*, and *ΔrpiAB Δapt* strains, and antibiotic susceptibility testing was performed.

### 2.6. RNA Extraction and qRT-PCR

*E. coli* MG1655, AM4001, AM4014 and AM4015 cells were grown until OD_600_ ≈ 0.5, mixed with ice-cold ethanol–phenol stop solution to inactivate cellular RNases, pelleted at 4 °C, and stored at −80 °C. Total RNA was extracted using the hot phenol method [18]. RNA quality was assessed by agarose gel electrophoresis and RNA concentration was determined spectrophotometrically (ND-100C, Changsha Nexisense Technology Co., Ltd., Changsha, Hunan Province, China). All RNA samples were treated with DNaseI (Fermentas, Vilnius, Lithuania) to remove contaminating genomic DNA. One microgram of total RNA after DNaseI treatment was reverse-transcribed with MMLV reverse transcriptase (Evrogen, Moscow, Russia) and specific oligonucleotide primers according to the manufacturer’s instructions. RT–qPCR was performed using qPCRmix-HS SYBR (Evrogen, Moscow, Russia) on a QuantStudio 5 Real-Time qPCR system (Applied Biosystems, Waltham, MA, USA). *rpoC* (RNA polymerase β′-subunit) served as the housekeeping gene. Fold changes in expression levels were calculated using the Pfaffl method.

### 2.7. ATP Level Measurement

The intracellular ATP level was determined by the luminescent method using the Luminescent ATP Detection Assay Kit (Abcam, ab113849, Cambridge, UK) on a Tecan Spark plate reader (Tecan Group Ltd., Männedorf, Switzerland). Bacterial cultures were grown in 5 mL of LB medium to an optical density OD_600_ ≈ 0.5. Cells were collected by centrifugation and quickly washed twice in 2 mL of saline. Further sample preparation and luminescence measurements were carried out in accordance with the manufacturer’s recommendations. The obtained values were related to the concentration of total protein in cell extracts, determined by the Bradford method.

### 2.8. Determination of PRPP-S Activity

PRPP-S activity was determined spectrophotometrically using the Precice PRPP-S Assay kit (Novocib, Lyon, France). Cells of strains MG1655 AM4001, AM4005, AM4011, and AM4016 were grown in 10 mL of LB medium to an OD_600_ ≈ 0.5. Cells were collected by centrifugation and the cell pellets were suspended in 1 mL of ddH_2_O. The cells were disrupted by sonication, and PRPP-S activity was determined in the extracts according to the manufacturer’s instructions. The obtained values were related to the concentration of total protein in cell extracts, determined by the Bradford method.

### 2.9. Statistical Analysis

The data are shown as mean ± standard deviation measures from triplicate values obtained from 3 independent experiments. The statistical difference between experimental groups was analyzed by one-way ANOVA with Tukey correction for multiple comparisons. Probability values (*p*) less than 0.05 were considered significant. Statistical analysis was performed using the GraphPad Prism 9.1.2 software (GraphPad Software Inc., San Diego, CA, USA).

## 3. Results

### 3.1. Activation of the Deo-Operon Expression in the rpiAB Mutant

Previously, we found that exogenous ribose had a toxic effect on the growth of the *ΔrpiAB* mutant [5]. Surprisingly, sensitivity to ribose can be suppressed by a mutation in the *deoB* gene, which encodes the enzyme phosphopentomutase responsible for the translocation of the phosphate group from position C1 to C5 in deoxyribonucleoside or ribonucleoside (Figure 1). We associated this suppressive effect with a limitation in the conversion of R1P to R5P, considering ribonucleoside catabolism as an additional source of R5P. This observation required further study of the impact of the *deo*-operon on the phenotypic expression of the mutant. Since the genes of the *deo*-operon are designed to degrade deoxyribonucleosides, we compared the effects of exogenous deoxy- and ribonucleosides on the mutant growth. As follows from the results presented in Figure 2, the addition of exogenous purine ribonucleosides adenosine and guanosine to the growth medium inhibited the growth of *rpiAB* mutants (Figure 2a,b). Importantly, the addition of purine deoxyribonucleosides, in contrast, stimulated the growth of the *ΔrpiAB* mutant (Figure 2a,b). Deoxyribonucleosides are the source of dR5P, which, with the participation of deoxyriboaldolase DeoC (Figure 1), is converted into GAP and acetaldehyde, contributing to glycolysis and the Krebs cycle [19]. Purine ribo- and deoxyribonucleosides are essentially competitive for the same enzymes, DeoD and DeoB. However, DeoB prefers deoxyribonucleosides. Thus, the addition of exogenous purine deoxyribonucleosides inhibits the catabolism of purine ribonucleosides, which leads to the production of R5P. Furthermore, the dR5P formed as a result of deoxyribonucleoside degradation is converted into GAP and acetaldehyde by the enzyme DeoC, serving as an additional carbon source for central metabolism and stimulating growth. The addition of the nitrogenous bases adenine and guanine without the ribose component improves the growth of *rpiAB* cells (Figure 2a,b), which emphasizes the interrelation between the toxic effect of exogenous ribonucleosides and the increase in the R5P pool.

These data indicate that the *deo*-operon genes are responsible for the biosynthesis of ribose-5 phosphate, which has a toxic effect on the growth of the *ΔrpiAB* mutant. Indeed, as shown in Figure 2c the transcription of the *deoA* and *deoB* genes is significantly increased compared to wild-type cells. It is known that dR5P, the main inducer of the *deo*-operon, interacts with the DeoR repressor, preventing its binding to the operator [11]. However, the P3 promoter of the *deo*-operon is induced by ribonucleosides independently of the DeoR repressor (Figure 1). R5P is a precursor for *de novo* inosine synthesis, which activates *deoBD* gene expression from the third promoter. DeoBD enzymes also mediate the catabolism of purine deoxyribonucleoside, resulting in the accumulation of dR5P. Thus, if not directly, then indirectly through inosine, the increased R5P level in the *rpiAB* mutant may lead to the induction of the *deo*-operon.

### 3.2. Identification of the P3 Promoter’s Impact on the Sensitivity Phenotype Formation of rpiAB to Nucleosides and Antibiotics

*deoD* and *deoB,* genes controlled by the P3 promoter (Figure 1), are responsible for purine nucleoside degradation. The P3 promoter region overlaps with the structural region of the *deoA* gene, which complicates its mutagenesis [8]. To examine the impact of P3 on the expression levels of *deoBD* genes and to remove the effect of promoter occlusion, a deletion of the P1-P2 promoter region was carried out. It was found that the *ΔrpiAB* mutant has increased expression of the *deoB* gene (Figure 3a), which is independent of the P1-P2 promoters located in the leader region of the *deo*-operon upstream of the *deoC* gene (Figure 3a). Furthermore, deletion of the P1-P2 promoters does not mitigate the toxic effects of adenosine on *ΔrpiAB* cells (Figure 3b) and does not suppress antibiotic sensitivity (Figure 3c,d).

Deletion of the *deoD* gene encoding purine nucleoside phosphorylase, as well as the deletion of the *deoB* gene encoding phosphopentomutase, which we revealed earlier [4,5], suppresses the sensitivity of the *rpiAB* mutant to antibiotics (Figure 4a,b). However, deleting the deoxyriboaldolase *deoC* gene does not affect the sensitivity of the *rpiAB* mutant (Figure 4a,b).

### 3.3. The Hypothetical Ribose-5-Phosphate Futile Cycle (RFC)

It is important to emphasize that, in addition to R1P formation, the DeoD enzyme activity produces purine bases (Figure 1). Purine bases are known to be involved in the so-called salvage pathway, which is carried out by phosphoribosyltransferase enzymes, resulting in the formation of nucleotide monophosphates. We demonstrated that a mutation in the *apt* gene, encoding adenine phosphoribosyltransferase, suppresses the increased antibiotic sensitivity of the *rpiAB* mutant (Figure 5a,b) and increases the ATP pool in the cells of this mutant (Figure 5c). Deletions of the *deoB* and *deoD* genes also increase ATP levels more than twofold compared to the original *ΔrpiAB* mutant (Figure 5c). The suppressive effects of *deoB*, *deoD* and *apt* mutations on antibiotic sensitivity and ATP levels can be reversed by introducing wild-type alleles of the corresponding genes (Figure 4a and Figure 5a,c).

Deletion of the *apt* gene significantly improves the growth of the *rpiAB* mutant and also eliminates the toxic effect of exogenous adenosine (Figure 5d). PRPP is a key metabolite of the salvage pathway, the level of which is controlled by the activity of the *prs* gene encoding PRPP synthase and the intracellular content of R5P. It should be noted that *prs* deletion is lethal for *E. coli* cells, complicating studies of its role in antibiotic sensitivity [15,20]. However, mutations that restrict adenine release (*deoD*) or adenine conversion to AMP (*apt*) lead to a decrease in Prs enzyme activity (Figure 5e).

In summary, it can be assumed that the *deoBD*, *apt* and *prs* genes together constitute a futile cycle (RFC). Futile cycles (FCs), also known as substrate cycles, are a pair of opposing biochemical reactions that continually convert a substrate into a product and then back. In doing so, FCs waste ATP without producing a tangible metabolic output [21]. A striking example of futile cycles associated with the loss of ATP is the work that shows a fundamental connection between ATP metabolism, endogenous ROS production, and tolerance toward oxidative stress in bacteria [22].

It is important to emphasize that in addition to AMP biosynthesis via the Prs-controlled reaction, high intracellular levels of PRPP are consumed in the *de novo* biosynthesis of AMP—a process on which the cell expends approximately 10 ATP molecules [23]. It has been shown previously that stimulation of purine biosynthesis by adding exogenous purine nucleotide precursors PRPP and glutamine enhances the bactericidal action of the antibiotics ampicillin and ciprofloxacin, which was associated with the consumption of ATP for the synthesis of purines *de novo* [24]. Therefore, operation of a futile cycle in the *rpiAB* mutant can result in considerable ATP consumption. As shown in Figure 6, AMP, which plays a central role in the cycle, is created during many anabolic processes, particularly in the reaction controlled by the *prs* gene. Intracellular AMP undergoes dephosphorylation to form the nucleoside adenosine, which in turn, with the participation of the enzyme purine nucleoside phosphorylase (DeoD), is degraded to adenine and R1P. In the next step, with the participation of the DeoB enzyme, R1P is transformed into R5P, a substrate for the Prs enzyme, which converts it to PRPP. It is important to emphasize that the latter reaction is accompanied by the consumption of ATP to produce AMP. Furthermore, the AMP pool can be replenished from adenine (the product of the DeoD reaction) and PRPP via the aforementioned salvage pathway involving the enzyme Apt.

## 4. Discussion

Recently, the exceptional importance of purine nucleoside metabolism in suppressing pathogen resistance to antibacterial drugs has been demonstrated [25,26]. Mutations affecting *de novo* purine biosynthesis and its regulation through the PurR repressor are associated with altered pathogen energy status and antibiotic tolerance [27]. In this study, we observed an increase in the expression of the *deo*-operon genes in *rpiAB* cells, which control nucleoside catabolism. Regarding the *rpiAB* mutant with a deleted promoter region P1-P2 of the *deo*-operon, we demonstrated that growth suppression by exogenous ribonucleosides is caused by the function of the internal promoter (P3), located upstream of the *deoBD* genes (Figure 1). We propose that the *deoBD* genes are involved in the creation of the hypothetical R5P futile cycle (RFC), which can be visualized in the *rpiAB* mutant. The main components of the RFC, in addition to the *deoBD* genes are the housekeeping gene *prs*, encoding PRPP synthesis, and the *apt* gene, responsible for AMP synthesis. These genes are participants in the so-called salvage pathway, which prevents purine nucleobases from degradation to uric acid. The creation of such a cycle is supported by the suppression of the *rpiAB* antibiotic sensitivity phenotype when the *apt* gene is damaged.

The result of the RFC functioning in the *rpiAB* mutant is a constantly occurring reaction of ATP pool depletion, thus limiting culture growth. One ATP molecule is converted to AMP and PPi per one round of the futile cycle:

ATP → AMP + PPi

We hypothesize that this mechanism of ATP pool reduction, triggered by high R5P levels in the *rpiAB* mutant cells, is one of the reasons for the increased sensitivity of this mutant to a broad spectrum of antibiotics.

Recent research contains contradictory evidence regarding ATP levels as a factor in antibiotic sensitivity/resistance. It is known that the non-specific action of bactericidal antibiotics, such as fluoroquinolones and aminoglycosides, involves the activation of cellular respiration, which is accompanied by an increase in the levels of ROS and ATP [28,29]. It has also been revealed that ATP release is a lethal factor associated with the depletion of divalent metal ions in the cell [29]. Additionally, high ATP levels are required for efficient transport and the creation of lethal intracellular concentrations of aminoglycosides [30,31]. On the other hand, low ATP levels are associated with ciprofloxacin persistence and tolerance in *E. coli* and other bacteria [32,33,34]. Furthermore, it has been shown that antibiotic resistance and persistence are regulated by the rates of ATP production and consumption, not its intracellular concentration [35]. Thus, we assume that ATP levels as a factor in antibiotic sensitivity/resistance should be considered in the context of the cell’s metabolic status.

The impact on cellular bioenergetics is one of the areas of development of new antimicrobial drugs aimed at combating resistant pathogens [36]. Within the context of the dependence of pathogen virulence on tolerance to oxidative stress, biochemical processes representing futile cycles of ATP hydrolysis were recognized and investigated in *E. coli* cells, including phosphoenolpyruvate carboxylase and phosphoenolpyruvate carboxykinase, the acetate cycle and the ATP synthase cycle. The proposed approach of inducing futile cycles can be used to enhance the sensitivity of bacteria to oxidants, which are important antimicrobial agents of the immune system [22]. The mechanism of non-specific action of beta-lactams on *E. coli* cells has been previously characterized as a futile cyclic process of cell wall synthesis and degradation [21].

Previously, we unexpectedly discovered that the *deoB* gene is required to maintain high sensitivity of the *E. coli rpiAB* mutant to a wide range of antibiotics [5]. However, it was unclear how the *deoB* gene was involved in regulating the mutant’s sensitivity. This study revealed that the expression of *deoBD* genes responsible for purine nucleoside degradation is coordinated with the activity of salvage pathway genes *apt* and *prs*. Based on the proposed model, these genes create a specific futile cycle of R5P which consumes ATP. According to the obtained experimental data, a correlation is observed between the ATP level in the *rpiAB* mutant and sensitivity to antibiotics and ribosides.

We hypothesize that the combination of decreased ATP levels and an enhanced level of R5P results in the hypersensitivity of the *rpiAB* mutant to various antibiotics. Confirmation of this hypothesis, based on genetic data, requires further research, including metabolic flux analysis.

## 5. Conclusions

In the context of recent research focusing on the role of bacterial metabolic status in antibiotic susceptibility, an approach based on the impact on cellular bioenergetics has acquired particular significance. The induction and cyclization of trivial metabolic processes related to housekeeping appear to be the most attractive and effective in terms of reducing the adaptive potential of bacterial cells and increasing the effectiveness of antibiotic therapy. In this study, based on genetic data, we propose a mechanism for ATP depletion by the induction of an ATP conversion cycle to AMP with the participation of the housekeeping gene *prs*, the *deo*-operon genes *deoBD* responsible for purine nucleoside catabolism, and the *apt* salvage pathway gene in the *rpiAB* mutant. It is important to emphasize that a key feature of the metabolic status of the *rpiAB* mutant is the one-way synthesis of R5P via the non-oxidative branch of the PPP, which leads to the accumulation of an intracellular pool of R5P. More specifically, the intracellular level of R5P determines the volume of the futile cycle, which we consider as a mechanism of cell de-energization and increased sensitivity to antibiotics.

## Figures and Tables

**Figure 1:**
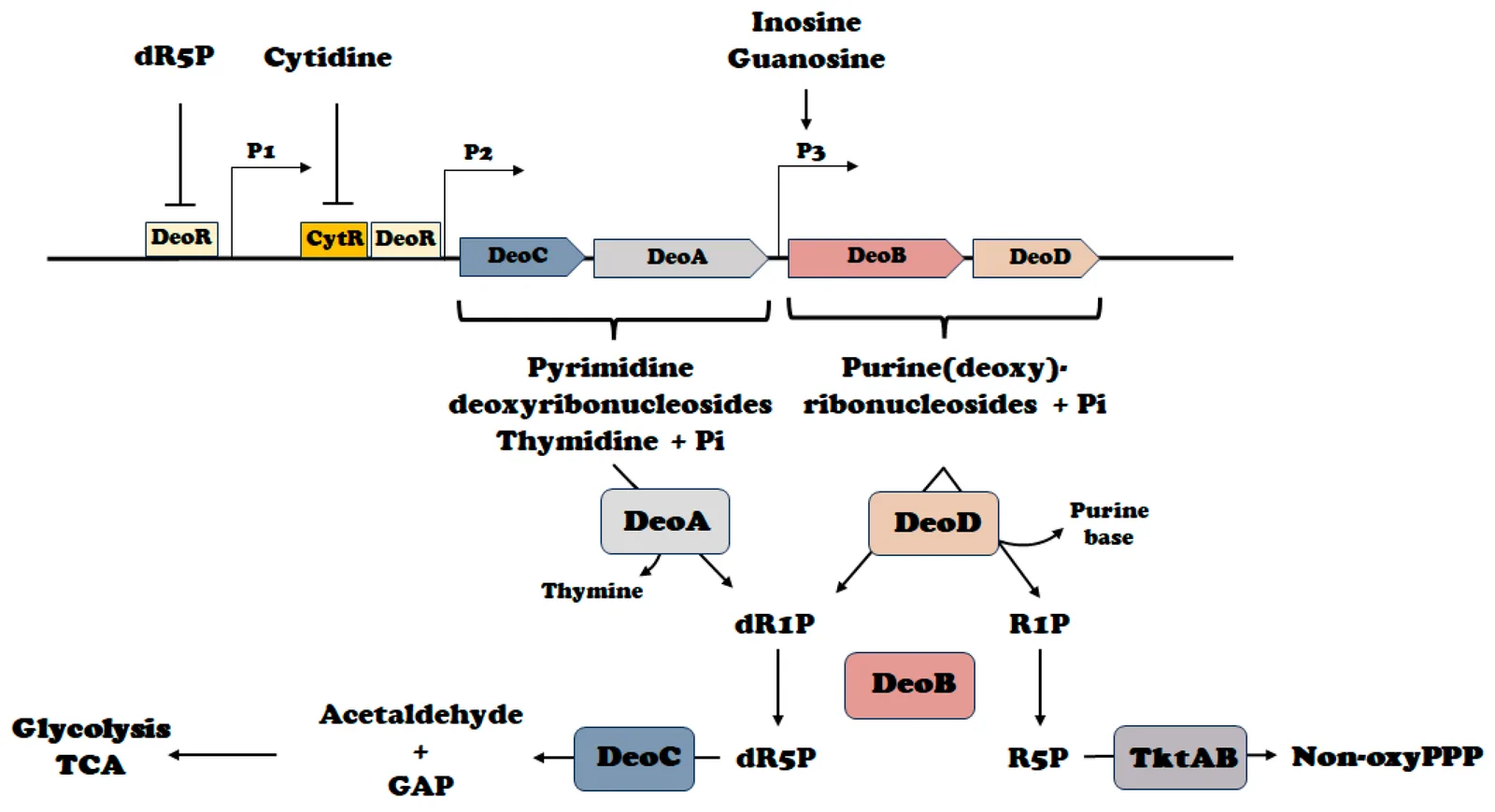
Scheme of the *deo*-operon and metabolic reactions of nucleoside catabolism in *E. coli* cells. The genes encoding the enzymes that degrade nucleosides constitute the *deo*-operon. The leader region of the *deoC* gene contains the P1 and P2 promoters, where the main transcriptional regulators are the repressors DeoR and CytR. Transcriptional inducers that prevent the binding of the repressors to the operator regions upstream of the *deoC* gene are deoxyribose-5-phosphate (dR5P), which interacts with DeoR, and cytidine, which interacts with CytR. The P3 promoter, which is activated by the purine ribonucleosides inosine and guanosine, located upstream of the *deoB* gene.

**Figure 2:**
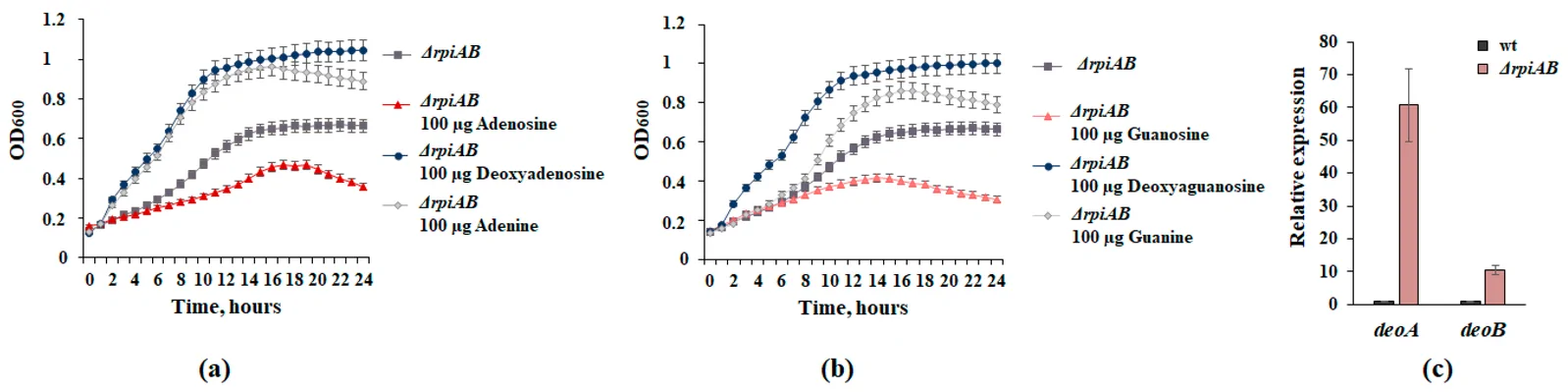
The impact of excess nucleosides on the growth of the *ΔrpiAB* mutant and the expression of the *deo*-operon. (**a**,**b**) Toxic effects of exogenous purine nucleosides adenosine and guanosine on the growth of the *ΔrpiAB* strain. The addition of deoxyribonucleosides, as well as the nitrogenous bases adenine and guanine, has a growth-stimulating effect on the *ΔrpiAB* strain. (**c**) Increased expression of *deoR*-regulated *deoA* and *deoB* genes in the *ΔrpiAB* mutant. Expression of *deoA* increases approximately 60-fold and *deoB* 10-fold compared to the parent strain. Relative expression was measured by RT-qPCR and normalized to *rpoC*. Mean values ± SD from at least three independent experiments are shown.

**Figure 3:**
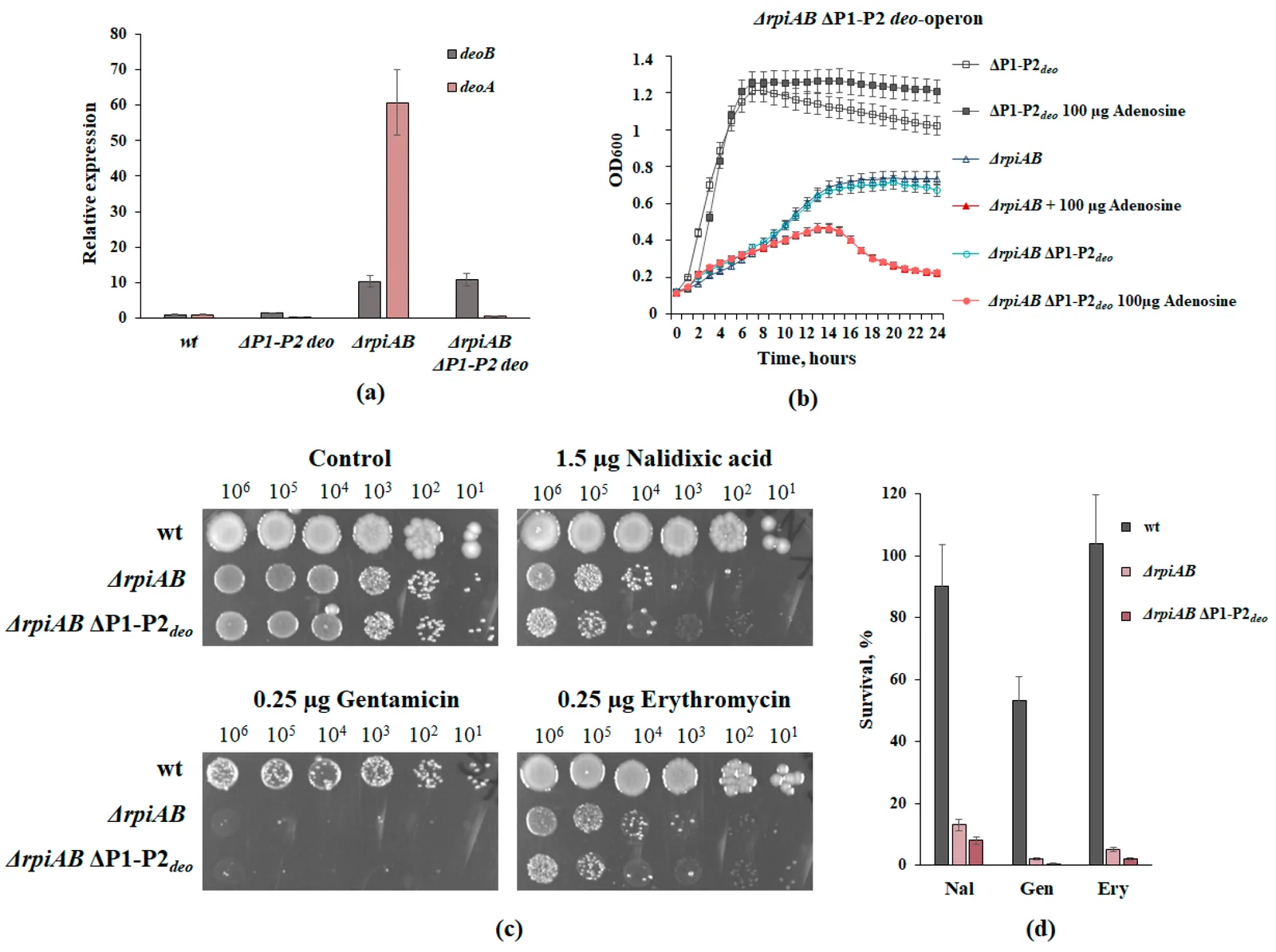
The deletion of the P1-P2 promoter region of the *deo*-operon does not affect the phenotype of the *rpiAB* mutant. (**a**) Expression levels of the *deoB* and *deoA* genes in the *ΔrpiAB* and *ΔrpiAB* ΔP1-P2 mutants. The ΔP1-P2 mutation inactivates *deoA* gene expression. The expression level of *deoB* does not change in the presence of P1-P2 deletion and remains 10-fold higher compared to the parental strain. Relative expression was measured by RT-qPCR and normalized to *rpoC*. (**b**) Growth curves of the *ΔrpiAB* and *ΔrpiAB* ΔP1-P2 mutants in the presence of 100 µg adenosine. (**c**) Sensitivity of the *ΔrpiAB* and *ΔrpiAB* ΔP1-P2 strains to different groups of antibiotics (nalidixic acid (Nal), gentamicin (Gen) and erythromycin (Ery)). (**d**) Cell viability in time-killing experiments was determined by counting cfu. Antibiotics were added for 30 min at concentrations of 25 µg/mL Nal, 3 µg/mL Gen, and 150 µg/mL Ery. Mean values ± SD from at least three independent experiments are shown.

**Figure 4:**
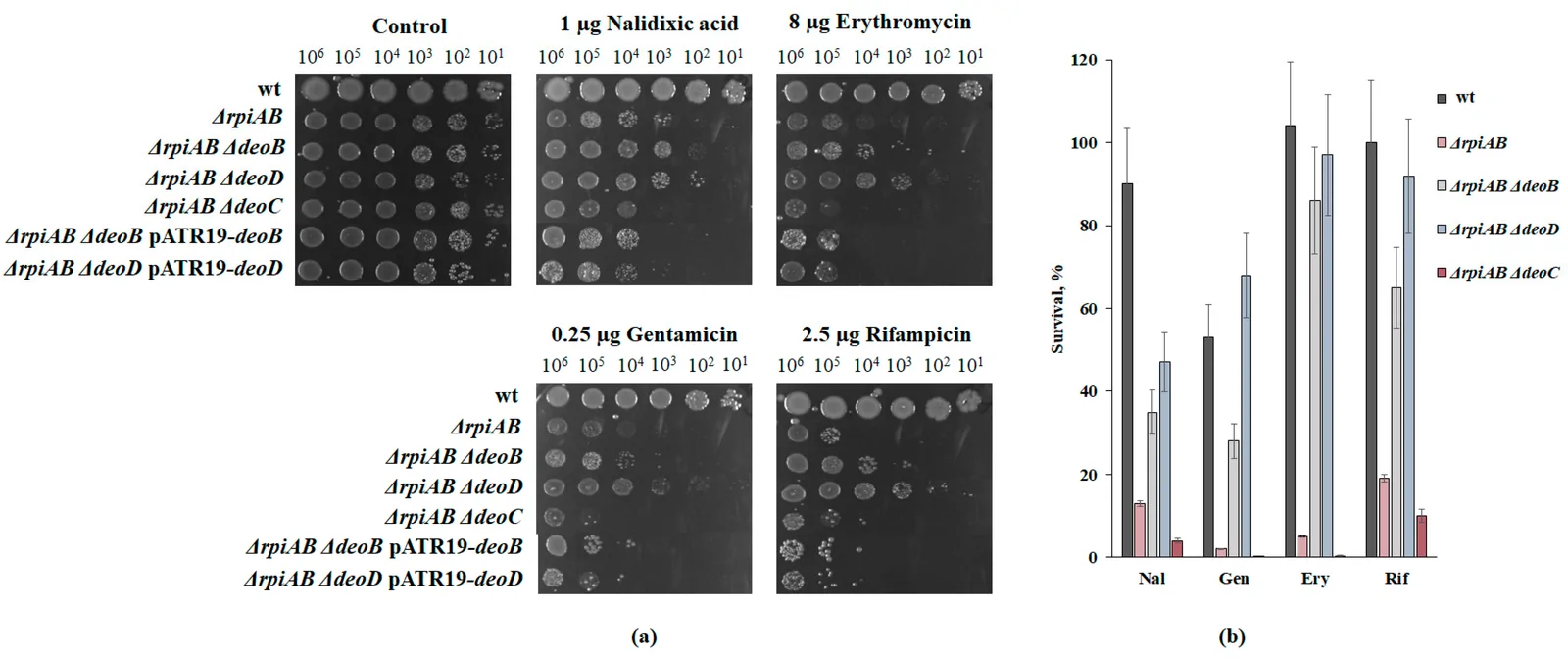
The effect of *deo*-operon gene deletions on the antibiotic sensitivity of the *ΔrpiAB* mutant. (**a**) Representative efficiencies of colony formation of WT (MG1655) and mutant *E. coli* cells in the presence of antibiotics: quinolones (nalidixic acid), aminoglycosides (gentamicin), macrolides (erythromycin), and inhibitors of RNA polymerase (rifampicin (Rif)). Introduction of plasmids carrying the structural regions of the wild-type *deoB* and *deoD* genes reverts the suppressive effect of *deoB* and *deoD* deletions in the *rpiAB* mutant. Ten -fold dilutions of bacterial cultures were placed on agar plates with the addition of appropriate concentrations of antibiotics and incubated overnight at 37 °C. (**b**) Cell survival in time-killing experiments was determined by counting cfu. Antibiotics were added for 30 min at concentrations of 25 µg/mL Nal, 3 µg/mL Gen, 150 µg/mL Ery and 25 µg/mL Rif. Mean values ± SD from at least three independent experiments are shown.

**Figure 5:**
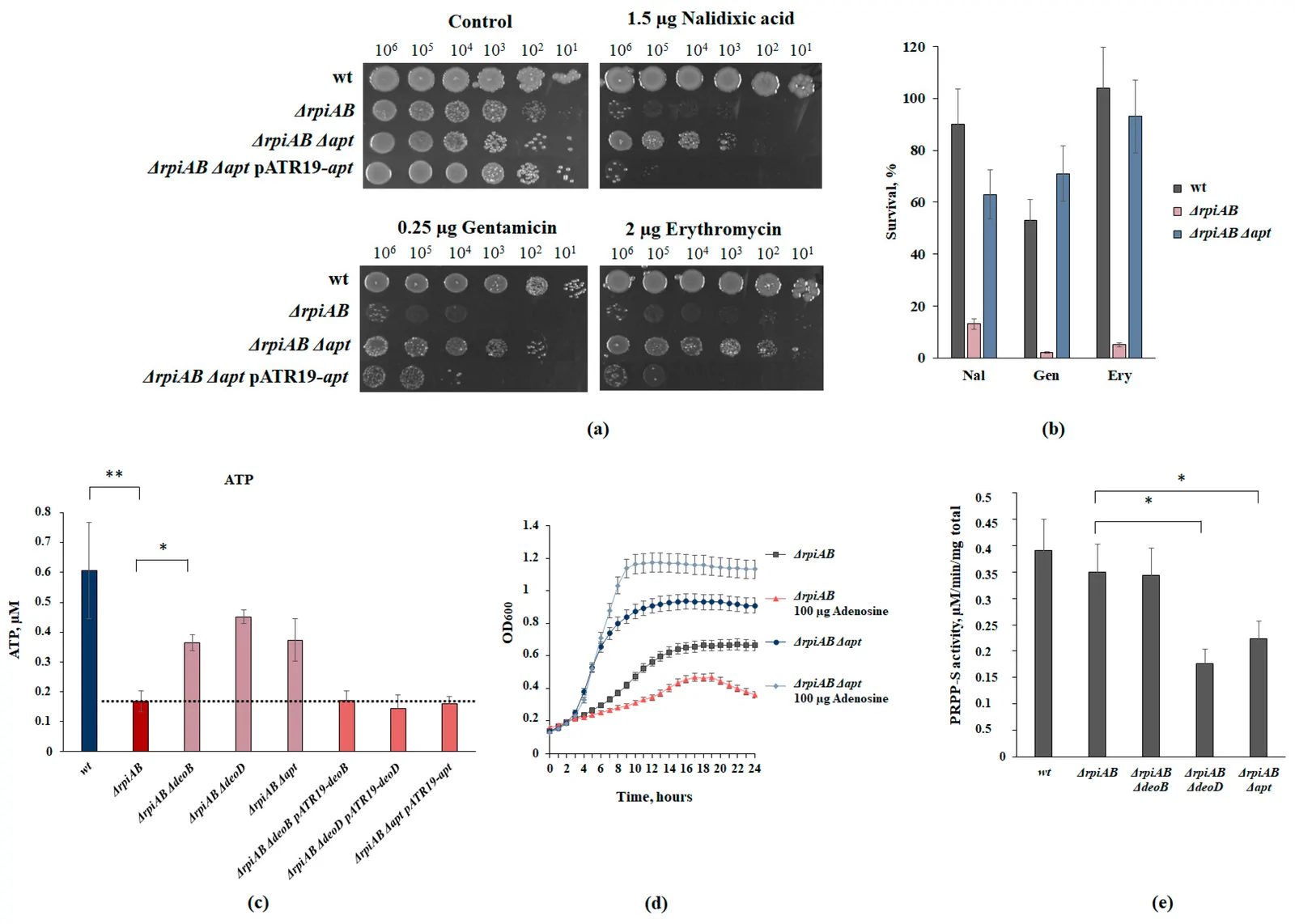
The role of the salvage pathway in the creation of the proposed futile cycle involving the *deo*-operon genes *deoD* and *deoB*. (**a**) Suppression of antibiotic sensitivity of the *rpiAB* mutant in the presence of deletion of the *apt* gene encoding adenine phosphoribosyltransferase. Plasmid insertion of the wild-type *apt* allele restores the antibiotic sensitivity of *rpiAB*. (**b**) Cell survival was determined by counting cfu. Overnight cultures of the indicated *E. coli* strains were diluted with fresh LB 1:100 and grown to OD_600_ ≈ 0.5. Antibiotics were added for 30 min at concentrations of 25 µg/mL Nal, 3 µg/mL Gen, and 150 µg/mL Ery. (**c**) Intracellular ATP level. The dashed line indicates the ATP level in the rpiAB mutant. * *p* < 0.05, compared to the *ΔrpiAB* cells. ** *p* < 0.01, compared to the wild-type cells. (**d**) The deletion of the *apt* gene suppresses the toxic effect of adenosine on the cells of the *rpiAB* mutant. (**e**) PRPP-S—ribose-phosphate pyrophosphokinase activity in cells of the *ΔrpiAB* mutant and triple mutants *ΔrpiAB ΔdeoB*, *ΔrpiAB ΔdeoD* and *ΔrpiAB Δapt*. * *p* < 0.05, compared to the *ΔrpiAB* cells. Mean values ± SD from at least three independent experiments are shown.

**Figure 6:**
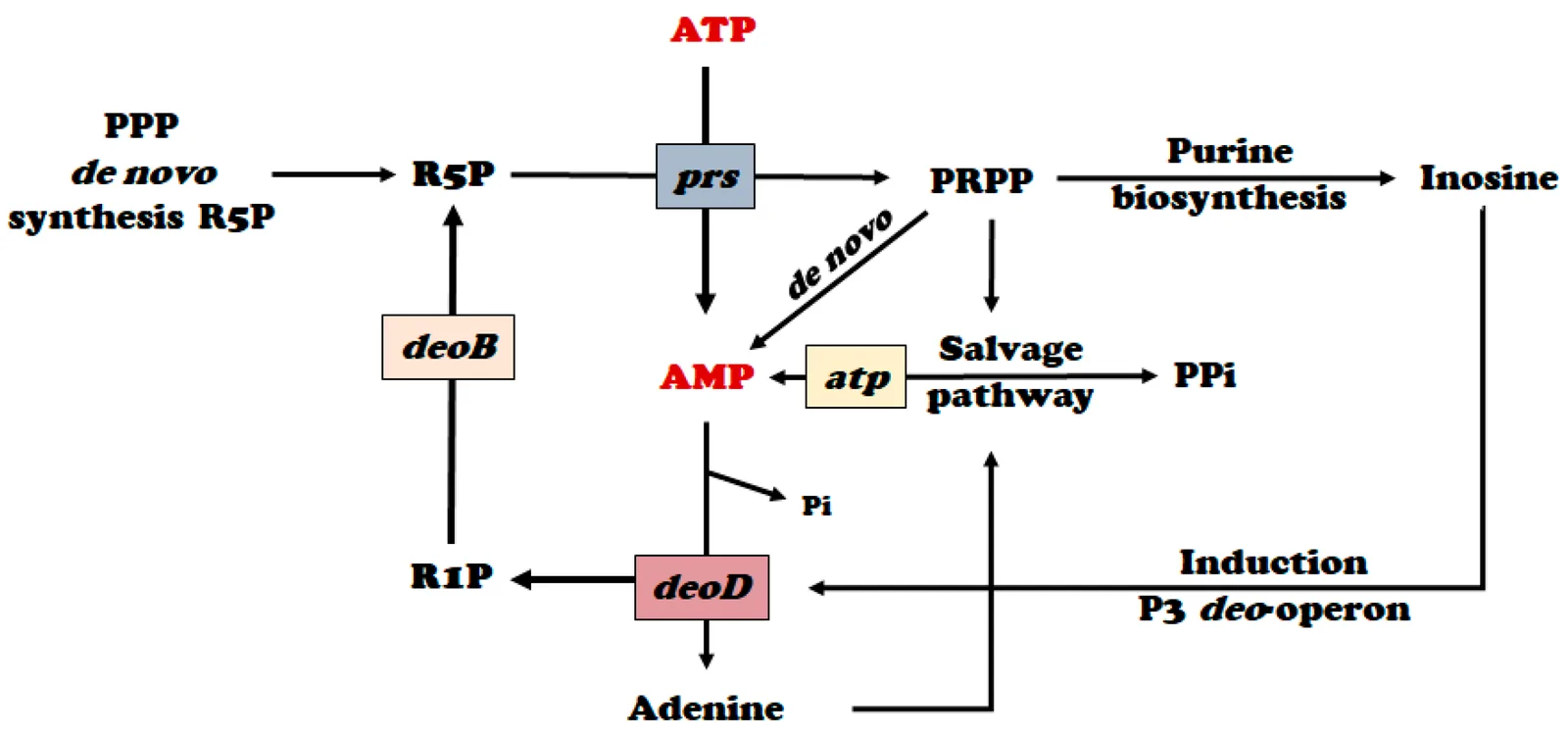
The hypothetical futile cycle (RFC) formed by the activities of the enzymes *prs*, *deoBD* and *apt*. Deprivation of ATP conditioned by activation of R5P in PRPP with the participation of ribose-phosphate pyrophosphokinase (Prs). RFC induced by increased levels of R5P in *ΔrpiAB* cells and coregulated via the cycling of R5P upon the destruction and resynthesis of the AMP molecule by enzymes of *deo*-operon DeoD, DeoB and adenine phosphoribosyltransferase Apt of the salvage pathway.

**Table 1:** Bacterial strains and plasmids.

Strain	Genotype	Reference
MG1655	F^–^ wild type	Laboratory collection
AM4001	As MG1655 plus *∆rpiAB*	[5]
AM4005	As AM4001 plus *∆deoB*	[5]
AM4011	As AM4001 plus *∆deoD*	This work
AM4012	As AM4001 plus*∆deoC*	This work
AM4014	As MG1655 plus *∆P1-P2_deo_*	This work
AM4015	As AM4001 plus *∆P1-P2_deo_*	This work
AM4016	As AM4001 plus *∆apt*	This work
**Plasmid**		
pUC19	AmpR, lac promoter	Laboratory collection
pATR-*deoB*	pUC19 carrying *deoB*	[4]
pATR-*deoD*	pUC19 carrying *deoD*	This work
pATR-*apt*	pUC19 carrying *apt*	This work

## Data Availability

The original contributions presented in the study are included in the article. Further inquiries can be directed to the corresponding author.

## Appendix A

**Table A1:** Primers used in this study.

Primer	Sequence
Del P1-P2 1	5′—atttcacgggggtattggcgacaaactccgccagttcgctcaagttagtataaaaaagct—3′
Del P1-P2 2	5′—cagtgcacgcaggctgcttgctttcagatcagtcattgaagcctgcttttttatactaag—3′
Del P1-P2 3	5′—actgtgcgtgtaacatggcg—3′
Del P1-P2 4	5′—gtcgtcattcagggtggtca—3′
Apt 1	5′—tacagccagtacattctggc—3′
Apt 2	5′—ggaaggtggattcacgaagg—3′
DeoB 1	5′—cggtatactgatctgatcatt—3 ’
DeoB 2	5′—ataacaaaggcggagtcaac—3′
DeoD1	5′—cgt tga ctc cgc ctt tgt ta—3′
DeoD2	5′—gat-act-aaa-aag-ccg-gag-cag—3′
DeoC1	5′—cta-aca-aac-tcg-caa-ggt-gaa—3′
DeoC2	5′—acg-ggt-aga-tac-gtc-tcg-ta—3′

**Table A2:** MICs for strains used in this study, mean values, µg/mL ± SD.

Strain	Nalidixic Acid (µg/mL)	Gentamicin (µg/mL)	Erythromycin (µg/mL)	Rifampicin (µg/mL)
MG1655	8.75 ± 0.25	1.25 ± 0.05	95 ± 2.5	13 ± 0.5
*ΔrpiAB*	4.5 ± 0.25	0.25 ± 0.05	20 ± 2.5	5 ± 0.5
*ΔrpiAB ∆P1-P2_deo_*	4.5 ± 0.25	0.25 ± 0.05	20 ± 2.5	5 ± 0.5
*ΔrpiAB ΔdeoB*	8 ± 0.25	1 ± 0.05	85 ± 2.5	12 ± 0.5
*ΔrpiAB ΔdeoD*	8.5 ± 0.5	1.25 ± 0.05	95 ± 2.5	13 ± 0.5
*ΔrpiAB ΔdeoC*	4.5 ± 0.25	0.25 ± 0.05	20 ± 2.5	5 ± 0.5
*ΔrpiAB Δapt*	8.5 ± 0.5	1 ± 0.05	85.5 ± 5	12.5 ± 0.5
